# *Lactobacillus fermentum* CECT5716 Supplementation in Rats during Pregnancy and Lactation Impacts Maternal and Offspring Lipid Profile, Immune System and Microbiota

**DOI:** 10.3390/cells9030575

**Published:** 2020-02-28

**Authors:** Ignasi Azagra-Boronat, Alba Tres, Malén Massot-Cladera, Àngels Franch, Margarida Castell, Francesc Guardiola, Francisco J. Pérez-Cano, Maria J. Rodríguez-Lagunas

**Affiliations:** 1 Physiology Section, Department of Biochemistry and Physiology, Faculty of Pharmacy and Food Science, University of Barcelona (UB), 08028 Barcelona, Spain; ignasiazagra@ub.edu (I.A.-B.); malen.massot@ub.edu (M.M.-C.); angelsfranch@ub.edu (À.F.); margaridacastell@ub.edu (M.C.); mjrodriguez@ub.edu (M.J.R.-L.); 2 Nutrition and Food Safety Research Institute (INSA-UB), 08921 Santa Coloma de Gramenet, Spain; atres@ub.edu (A.T.); fguardiola@ub.edu (F.G.); 3 Department of Nutrition, Food Science and Gastronomy, Faculty of Pharmacy and Food Science, Torribera Food Science Campus, University of Barcelona (UB), 08921 Santa Coloma de Gramenet, Spain

**Keywords:** probiotic, gestation, breastfeeding, rodents, fatty acids

## Abstract

Probiotics have shown potential for their use in early life. This study aimed to investigate whether the administration of *Lactobacillus fermentum* CECT5716 during pregnancy and lactation periods impacts maternal and offspring plasma lipid profile, immune system and microbiota. Rats were supplemented with the probiotic during gestation and two weeks of lactation. After supplementation, although the microbiota composition was not affected, the probiotic strain was detected in all cecal contents of dams and in some of their pups. Dams showed reduced proportion of T cytotoxic cells in the mesenteric lymph nodes, modulation of intestinal cytokines (IL-10 and IL-12) and changes in plasma fatty acids (20:0, 22:0, 20:5 *n-3*, and 18:3 *n-6*). Pups showed changes in immunoglobulins (intestinal IgA and plasmatic IgG2a and IgG2c) and fatty acid profile (17:0, 22:0, and 18:2 *n-6*). Overall, *Lactobacillus fermentum* CECT5716 supplementation contributed to beneficially modulating the immune system of the mother and its offspring.

## 1. Introduction

Pregnancy and breastfeeding are critical periods in which maternal diet plays a pivotal role in the correct development and growth of the fetus and infant [1]. These stages comprise important physiological changes that affect the microbiota of the mother and, therefore, the acquisition of the microbiota in the offspring.

Firstly, the vaginal microbiome of the mother has been shown to change during pregnancy, by decreasing α diversity and expanding *Lactobacillus* species, probably with the aim of maintaining a low pH that protects the fetus from infection [2,3]. In addition, the maternal gut microbiota increases the abundance of Actinobacteria and Proteobacteria and decreases that of *Faecalibacterium* in the third trimester of pregnancy [2,4], thus achieving a microbiota far more pro-inflammatory than that found in nonpregnant individuals [5]. These changes resemble those found in metabolic syndrome and might be beneficial to pregnancy by the acquisition of a microbiota with higher energy-yielding capacity and able to promote the physiological insulin resistance to ensure appropriate energy transfer to the fetus [2,3,4]. In turn, this changed microbiota composition is also shaped in order to provide an initial inoculum to the newborn after delivery [6]. 

Nevertheless, there is accumulating evidence that the fetus already develops an initial microbiome in utero before birth [7,8]. Distinct microbial communities have been detected in the placenta and amniotic fluid and may be transferred to the fetal gastrointestinal tract by swallowing the amniotic fluid [9,10]. The precise route of maternal transmission is still not clear, but might include colonization of the placenta by bacteria traveling through the bloodstream or by bacteria ascending the vagina [2].

At birth, vaginally delivered infants receive the initial inoculum when descending the birth canal. This acquisition is characterized by *Bifidobacterium* and *Streptococcus* species. In contrast, the gut of infants delivered by caesarean section is colonized with less bifidobacteria and more *Clostridium* and *Bacteroides* [11,12,13]. Thereafter, breastfeeding is one of the major contributors to the development of the microbiota of the gastrointestinal tract. On the one hand, human milk contains a high abundance of undigestible oligosaccharides that shape the intestinal microbiota by favoring the proliferation of certain bacteria, such as *Bifidobacterium* spp. [3,14] On the other hand, the translocation of bacteria from the gastrointestinal tract of mothers to the mammary gland (known as the entero-mammary route) has been suggested as a mechanism to explain the great increase of bacteria in the mammary tissue during lactation [15,16]. Indeed, milk microbiota has also been identified as a continuous source of bacteria to the newborn; a baby consuming about 800 mL of breast milk daily will ingest 10^5^–10^7^ bacteria/day [16,17]. 

The immune system also undergoes alterations in the perinatal period. Maternal hormones induce changes to achieve tolerance towards and adequate support for the fetus. For most of the pregnancy period a Th2-type response is promoted with the aim of sustaining fetal tolerance. Implantation and ejection are the only phases orchestrated by pro-inflammatory responses [3]. After delivery, the immune system of the newborn is still immature, and breastfeeding reinforces its development and helps to prevent and fight infections. In this context, breast milk contains immune factors, such as immunoglobulins (Ig), cytokines and immune cells [18]. Moreover, breast milk also contains multiple other bioactive factors that influence directly or indirectly the immune system of the newborn, such as antimicrobial components, growth factors or long-chain polyunsaturated fatty acids (LC-PUFAs) [19,20]. Compared to adults, newborns display reduced responsiveness of phagocytes and antigen-presenting cells and have more natural killer (NK) cells, but with weaker cytotoxic activity. In general, infants produce less Th1-type cytokines, more Th17-type cytokines, and more IL-10, and as their immune system matures, their response is biased from Th2 towards Th1 [9,21]. 

In this context, the administration of probiotics to mothers during pregnancy and breastfeeding periods has been considered a window of opportunity to prevent and reduce the risk of several diseases in either the mother or the offspring. There is evidence that probiotic supplementation in mothers during gestation and breastfeeding reduces the risk of eczema in the infants, and that such an approach is as effective as administering the probiotics directly to the infant [22,23,24,25,26,27]. However, current evidence does not indicate that probiotics can reduce the development of other allergic diseases, such as asthma [28,29]. Several studies also found positive outcomes in the risk of gestational diabetes mellitus [30,31,32], vaginal dysbiosis [33,34,35,36], preeclampsia [37,38], or preterm delivery [39]. 

Amongst probiotics, Lactobacillus are one of the most commonly used in early life. In particular, *Lactobacillus fermentum* CECT5716 is a probiotic strain isolated from breast milk [40] with proven safety and tolerance in infants and mothers [41,42,43]. In addition, this probiotic has been reported to produce beneficial outcomes during the perinatal period, for example by reducing gastrointestinal and upper respiratory tract infections in infants [44] or by becoming an effective alternative for the treatment of infectious mastitis during lactation [41,45,46]. Finally, *Lactobacillus fermentum* CECT5716 has shown a high immunomodulatory potential in vitro and in vivo [47,48,49]. 

Previous studies in our laboratory evidenced that the supplementation with this probiotic strain in rats during pregnancy and breastfeeding periods was able to positively modulate the breast milk composition of fatty acids (FAs) and Ig by increasing its content of IgA and PUFAs [50]. In addition, the probiotic was able to reach in the breast milk of some individuals. However, it is unknown whether this impact on the breast milk composition might arise from changes in the gut and systemic compartments of the mother and whether these outcomes, in turn, can impact their offspring. Therefore, the objective of the present study was to investigate whether the administration of *Lactobacillus fermentum* CECT5716 during pregnancy and lactation periods impacts the maternal and offspring plasma lipid profile, immune system, and microbiota composition.

## 2. Materials and Methods

### 2.1. Animals

Wistar adult rats (18 females and 3 males, RjHan:WI) were obtained from Janvier Labs (Le Genest-Saint-Isle, France) and individually housed in cages containing large fibrous-particle bedding. Dams were given a standard diet corresponding to the American Institute of Nutrition 93G formulation [51] (Teklad Global Diet 2014, Envigo, Indianapolis, IN, USA) and water ad libitum. An acclimatization period of 7 days was established in order to allow the animals to stabilize in the new environment. Then, animals were crossed by placing two randomly selected females inside the cage of each of the males for 24 h. After crossing (G0), 12 pregnant dams were individually housed, monitored daily and allowed to deliver naturally. The day of birth was established as day 1 of life (L1). On day 2, litters were culled to 8 pups per lactating dam, with free access to maternal milk and rat diet. Animals were housed under controlled conditions of temperature and humidity in a 12 h light–12 h dark cycle, in the Faculty of Pharmacy and Food Science animal facility (University of Barcelona, Spain). 

All experimental procedures were conducted in accordance with the institutional guidelines for the care and use of laboratory animals and were approved by the Ethical Committee for Animal Experimentation of the University of Barcelona and the Catalonia Government (CEEA-UB Ref. 486/16 and DAAM9268, respectively), which are in full compliance with national legislation following the EU-Directive 2010/63/EU for the protection of animals used for scientific purposes.

### 2.2. Experimental Design 

Animals were randomly divided into two groups (REF and LF) consisting of six dams per group and their respective eight pups (*n* = 48 pups per group). Dams in the LF group were daily supplemented with 10^10^ UFC/day of Lactobacillus fermentum CECT5716 (LF, kindly provided by Biosearch S.A., Granada, Spain) by oral gavage for 5 weeks, corresponding to the 3 weeks of gestation (from G0 to G21) and the first 2 weeks of lactation (from L1 to L14). The last day of sampling (L14) was considered free of the influence of coprophagy because animals do not start ingesting maternal feces until L16 [52]. The reference (REF) group included six dams receiving the vehicle (maltodextrins in water) in the same conditions. Feces from dams were collected weekly both during gestation (G0, G7, G14, G21) and lactation (L7 and L14). At the end of the intervention (L14, 14 days after birth), dams (*n* = 6/group) and pups (*n* = 48/group) were anesthetized with ketamine (90 mg/kg) and xylazine (10 mg/kg) (Bayer A.G., Leverkusen, Germany). Organs were collected for weight assessment of dams and pups (stomach, small intestine, cecum, colon, spleen, liver, and kidneys) and others additionally of dams (duodenum, jejunum, ileum, uterus, ovaries, and heart). Moreover, mesenteric lymph nodes (MLNs) of dams were obtained to assess the proliferation and the proportion of lymphocyte cell populations and to quantify IgA and IgM.

An aliquot of whole blood of dams was collected in heparin tubes for the analysis of the phagocytic activity of granulocytes and monocytes. Moreover, a second aliquot of blood of all animals was collected in EDTA tubes and then centrifuged in order to obtain plasma. Plasma samples were used for the determination of Ig, cytokines and FA profile. The cecal content (CC) was kept for the analysis of the microbiota composition and for tracking the probiotic. The small intestine was opened lengthwise, cut into 5 mm fragments and incubated with 20 mL (dams) or 2 mL (pups) of PBS in a shaker (10 min, 37 °C) to obtain the gut wash (GW). Feces, GW and submaxillary gland (SMG) of dams were obtained for the quantification of IgA and IgM. All samples were stored at −80 °C.

Weight was monitored weekly in dams, both during gestation (G0, G7, G14, G21) and lactation (L7 and L14), and daily in pups. Moreover, at the end of the study (L14) morphometric variables were analyzed: The naso–anal and tail lengths were measured to determine the body/tail ratio, the body mass index (BMI), which was calculated as body weight/length^2^ (g/cm^2^) and the Lee index, which was calculated as (weight^0.33^/length) × 1000 (g^0.33^/cm).

### 2.3. Extraction, Methylation, and Quantification of FA

The quantification of FAs was performed as in previous studies [50]. Briefly, the lipid fraction of plasma samples of dams and pups was extracted with a mix of CHCl_3_/MeOH 2:1 (*v*/*v*), and then derivatized to obtain the FA methyl esters (FAMEs), which were determined by gas chromatography in an Agilent 4890D chromatograph (Agilent Technologies, Waldbronn, Germany) equipped with an SP-2380 capillary column (60 m, 0.25 mm i.d., 0.2 µm, Supelco, Bellefonte, PA, USA), a split/splitless injector set at 270 °C and a flame ionization detector set at 300 °C. The split ratio was 1:30. For FAME separation the oven program was configured as follows: initial temperature 150 °C (held at this temperature for 1 min) up to 180 °C at 3 °C/min, from 180 °C (0.5 min) up to 220 °C at 14.5 °C/min, and from 220 °C (3 min) up to 250 °C at 9.9 °C/min, maintaining 9.5 min at 250 °C. The carrier gas was hydrogen (1.72 × 10^5^ Pa). Two µL of the samples were injected, and the FA were identified by comparing the retention time with a standard mix (Supelco 37 component FAME Mix, Sigma-Aldrich Co., St Louis, MO, USA). A total of 26 FAs were identified. The quantification was performed by peak area normalization (the quantitative results are obtained by expressing the area of a given peak as a percentage of the sum of the areas of all the identified peaks).

### 2.4. Quantification of Immunoglobulins and Cytokines

The quantification of Ig (IgM, IgG1, IgG2a, IgG2b, IgG2c, and IgA) and cytokines (IFNγ, TNFα, IL-4, IL-6, IL-10, and IL-12) in plasma of pups and in plasma of dams was performed at the end of the study, as previously described [53]. Briefly, specific color-coded capture beads were bound to the analyte of interest. Then, different detection antibodies conjugated to phycoerythrin (PE) were added for the detection of immunoglobulins. For the analysis of cytokines, antibodies were conjugated to biotin and afterwards incubated with streptavidin-PE. The specific concentration of each analyte was obtained by MAGPIX^®^ analyzer (Luminex Corporation, Austin, TX, USA) at the Cytometry Service of the Science and Technology Centers of the University of Barcelona (CCiT-UB). Assay sensitivity was as follows: 0.02 ng/mL for IgM; 0.78 ng/mL for IgG1; 0.02 ng/mL for IgG2a; 0.11 ng/mL for IgG2b; 0.19 pg/mL for IgG2c; 0.48 pg/mL for IgA; 3.5 pg/mL for IL-1α; 0.1 pg/mL for IL-4; 1.9 pg/mL for IL-6; 1.6 pg/mL for IL-10; 5.9 pg/mL for IL-12p70; 0.6 pg/mL for IFN-γ; and 0.4 pg/mL for TNF-α.

### 2.5. Analysis of Microbiota Composition

Genomic DNA was extracted from randomly selected CC (*n* = 3/group) of mother–pup pairs using the DNeasy Blood and Tissue Mini Kit (Qiagen, Madrid, Spain) and amplified following the 16S Metagenomic Sequencing Library Illumina 15044223 B protocol (Illumina Inc., San Diego, CA, USA), as previously described [54]. Sequenced data were available within approximately 56 h. Image analysis, base calling and data quality assessment were performed in the MiSeq instrument. The software Paired-End read merger (PEAR v0.9.6, Exelixis Lab, Heidelberg, Germany) was used to merge raw sequences forward and reverse. Sequences were trimmed, filtered and inspected for chimera, as previously described [54].

The qualitative presence or absence of genera was represented in a Venn diagram. A bacterial group was considered as present by establishing a cutoff of two animals (out of the three samples analyzed) displaying proportions higher than 0.001%. The list of genera beside each graph highlight those genera meeting the criteria that in two or three animals the detection of that genera is positive in one group while no animals display detection in the other group.

### 2.6. Phagocytic Activity of Blood Monocytes and Granulocytes

The phagocytic activity of blood monocytes and granulocytes of dams was determined with Phagotest^TM^ (Glycotope Biotechnology GmbH, Heidelberg, Germany), according to the manufacturer’s instructions and as previously described [55]. Briefly, heparinized blood was mixed and incubated at 37 °C with Escherichia coli opsonized with antibodies conjugated to fluorescein isothiocyanate (FITC). This allowed monocytes and granulocytes to phagocyte them. A control incubated at 0 °C was also included (no phagocytosis should be observed). Remaining unphagocyted bacteria were inactivated by adding a FITC-fading agent. Samples were cleaned, lysed, fixed, and incubated with a DNA staining. Finally, a CyAn ADP cytometer (Beckmann Coulter, Brea, CA, USA) was used to acquire fluorescence. The FITC (FL1) fluorescence was measured upon filtering with the DNA positive staining (FL3). The data obtained were analyzed with FlowJo v10.0.7 software (Tree Star Inc., Ashland, OR, USA). The percentage of phagocytosis (FL3+ FL1+) was calculated in the gate of monocytes and granulocytes and the median fluorescence intensity (MFI) was obtained in order to provide insight regarding the mean number of bacteria phagocyted.

### 2.7. Lymphocyte Isolation from Mesenteric Lymph Nodes (MLNs)

Cells were isolated from the MLNs of dams by passing the tissue through a 40 µm mesh cell strainer (Thermo Fisher Scientific, Barcelona, Spain). Then, the cell suspension was centrifuged (538× *g*, 10 min, 4 °C) and resuspended in Roswell Park Memorial Institute (RPMI) 1640 medium (Sigma-Aldrich, Madrid, Spain) enriched with 10% fetal bovine serum (FBS; Sigma-Aldrich), 100 IU/mL streptomycin–penicillin (Sigma-Aldrich), 2 mM L-glutamine (Sigma-Aldrich) and 0·05 mM 2-β-mercaptoethanol (Merck Millipore), as in previous studies [56]. CountessTM Automated Cell Counter (InvitrogenTM, Thermo Fisher Scientific) was used for cell counting and the assessment of viability. Lymphocytes were immediately used to assess their phenotype, proliferation and ability to secrete cytokines.

### 2.8. Immunofluorescence Staining and Phenotype of MLN Lymphocytes

MLN lymphocytes (3 × 10^5^ cells) were stained using immunofluorescence techniques, as previously described [57]. The mouse anti-rat monoclonal antibody (mAb) conjugated to FITC, PE, peridinin chlorophyll protein (PerCP), allophycocyanin (APC) or Brilliant Violet (BV) used here included anti-CD4 (OX-35), anti-CD8α (OX-8), anti-TCRαβ (R73), anti-TCRγδ (V65) and anti-NKR-P1A (10/78), all from BD Pharmingen (San Diego, CA, USA); anti-CD45RA (OX-33) from Caltag (Burlingame, CA, USA); anti-CD8β (3·41) from Serotec (Kidlington, Oxford, UK); anti-αE integrin (OX-62) and anti-CD62L (OX-85) from BioLegend (San Diego, CA, USA). Cells were incubated with a mixture of saturating concentrations of mAb in a phosphate-buffered saline solution containing 2% FBS and 0.1% NaN_3_ (Merck), at 4 °C in darkness for 20 min. After washing, cells were fixed with 0.5% p-formaldehyde (Merck) and stored at 4 °C in darkness until analysis by flow cytometry. A negative control staining using an isotype-matched mAb was included for each sample. Results were analyzed using a Gallios^TM^ flow cytometer (Beckman Coulter Inc., Madrid, Spain) in the cytometry service of the CCiT-UB. The obtained data were assessed with FlowJo v10.0.7 software (Tree Star Inc.).

### 2.9. Proliferative Response of MLN Lymphocytes

The T-lymphocyte proliferative response was assessed in MLN lymphocytes of dams, as in previous studies [58]. Ninety-six-well plates were coated with anti-CD3/anti-CD28 mAb (10 and 20 µg/mL, respectively; BD Biosciences). MLN lymphocytes (10^5^/200 µL) were incubated in quadruplicate with or without stimulus for 24 h. The BrdU Cell Proliferation Assay Kit (Merck Millipore) was used to quantify proliferation, following manufacturer’s instructions. Briefly, 5-bromo-2’-deoxyuridine (BrdU) was added to the cells and incorporated into the DNA while proliferating. Cells were washed, fixed and their BrdU content was determined through enzyme-linked immunosorbent assay (ELISA). After stopping the enzymatic reaction, the absorbance was measured at 450 nm on a microplate photometer (Labsystems Multiskan MS). The proliferation rate was expressed as the absorbance of the stimulated wells divided by the absorbance of the non-stimulated wells.

### 2.10. Quantification of Immunoglobulins in Different Compartments

The concentration of immunoglobulins in feces, GW, MLN, and SMG was quantified using a sandwich ELISA technique with the Rat IgM, IgG, and IgA ELISA quantification test from Bethyl Laboratories (Montgomery, TX, USA), as previously described [59]. Briefly, 96-well plates (Nunc MaxiSorp, Wiesbaden, Germany) were coated with 2 µg/mL of the capture antibody. After blocking, the standard and the samples were incubated. Finally, an adequate dilution of the peroxidase-conjugated detection antibody was added and, after washing, an o-phenylenediamine dihydrochloride-H_2_O_2_ (Sigma-Aldrich) solution was added. Absorbance was measured in a microplate photometer (LabSystems Multiskan) and data were interpolated by Multiskan Ascent v2.6 software (Thermo Fisher Scientific SLU, Barcelona, Spain) according to the concentration of the standard. Because this kit was not validated for samples other than plasma or serum, a recovery study was performed in adult and infant GW and in adult MLN, SMG and fecal homogenates, following immunoassay validation guidelines [60]. For that, the percentage of recovery after spiking these samples with the standard was calculated. In all cases, the recovery was between 82–105%, evidencing low matrix effect and good accuracy of the measurement (Supplementary Table S1).

### 2.11. Detection of Lactobacillus fermentum CECT5716

Detection and quantification of the probiotic strain in CC and fecal samples was performed by PCR techniques as described in Gil-Campos et al. 2012 [43]. The samples were weighed and homogenized in a FastPrep-24 instrument (MP Biomedicals, Inc., Santa Ana, CA, US) for 40 s. The DNA was obtained using the FastDNA kit (MP Biomedicals). The probiotic genome was detected following a nested PCR-based strategy. We performed a first standard amplification using the following oligonucleotides as primers: HSL40_126D (5′-GCTTGCCGCTTCTCTGGT-3′) and HSL40-126 (5′-CAACGACGATGAACACCACTT-3′) at 500 nM. The PCR conditions were an initial denaturing step for 5 min at 95 °C, followed by 40 cycles at 95 °C for 30 s, 46 °C for 30 s, and 72 °C for 30 s, and a final extension for 3 min at 72 °C. The result of the amplification was an amplicon of 222 base pairs. The second amplification was a Taq-Man-based PCR assay, the target sequence of which was located within the product of the first PCR. The forward primer was 5’-TCAACGGCCCCTTCAATACA-3’, the reverse primer used was 5’-GACCTAATTCACGTCAAACATATTTCAC-3’ and the probe labeled with VIC and CSY was 5’-AGTGGTGAGATGCCCAGTGT TCCCG-3’. Quantitative PCR assays were performed in duplicate for each sample using an ABI PRISM7700 Sequence Detection System (Applied BioSystems, Foster City, California, United States) in the CCiT-UB. A standard curve of the DNA from the probiotic strain in ranging dilutions 1/1 to 1/10^11^ was used to establish the cutoff of positive detection.

### 2.12. Statistical Analysis

The Statistical Package for the Social Sciences (SPSS v22.0) (IBM, Chicago, IL, USA) was used for the statistical analysis. Homogeneity of variance and normality distribution were tested by the Levene’s and Shapiro–Wilk tests, respectively. When data were homogeneous and had a normal behavior, Student t-test was used to analyze statistical differences. Otherwise, the Mann–Whitney U test was performed. Significant differences were established when *p* < 0.05. For the correlation of IgA, the Spearman’s correlation coefficient was calculated.

The fold change was calculated to analyze Ig, cytokines, and FAs by dividing individual values of the LF group by the mean of the values in the REF group. The results were subtracted from 1 and the mean value was calculated to obtain the mean fold change. Therefore, positive values indicate higher abundance in the LF group as compared to the REF group and negative values indicate lower abundances in the LF group as compared to the REF group.

A principal components analysis (PCA) was calculated with the plasma FA data using SIMCA v13.0 software (Umetrics, Umeå, Sweden). Two data matrices were constructed consisting of 11 rows and 20 variables for the dams and 48 rows and 21 variables for the pups. PCA was conducted on both data matrices in order to explore the presence of any natural clustering in the data and to search for outlier samples. Finally, two samples of pups’ plasma were detected as outliers and discarded from the study. To develop the model, FA data was mean-centered and scaled to unit variance.

## 3. Results

### 3.1. Transmission of Lactobacillus fermentum CECT5716

After the administration of the probiotic to the dams during the pregnancy period and 2 weeks of lactation, the detection of the specific strain was performed in the CC of dams and pups (Table 1). All the CC of the dams displayed positive detection of the probiotic. Furthermore, three of the dams displayed positive detection in the CC of 25–50% of their pups.

### 3.2. Growth, Morphometrics

The body weight, BMI and Lee index measures of dams and pups were not modified due to the probiotic supplementation (Table 2). Moreover, the organ weights and lengths of dams at the end of the study (L14) were similar in the REF and LF groups. Although we did not detect any significant changes in the organ weights and lengths of the pups, we detected a tendency towards an increase in brain weight (*p* = 0.074) and towards a decrease in liver weight (*p* = 0.051).

### 3.3. Impact on the Dams’ Immune System

**Figure 1:**
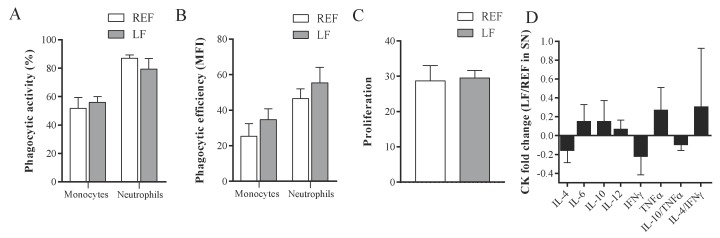
Systemic and intestinal immune cell function of dams at L14. The (**A**) phagocytic activity and (**B**) phagocytic efficiency of blood monocytes and neutrophils was measured. Moreover, (**C**) the proliferation of mesenteric lymph node lymphocytes was assessed after stimulation with CD3/CD28, and (**D**) cytokines in the supernatants were quantified by Luminex Technique. Results are expressed as mean ± S.E.M. (*n* = 6/group). The fold change was calculated with respect to REF group. REF, reference group; LF, *Lactobacillus fermentum* CECT5716 group; MFI, median fluorescence intensity; St., stimulated; CK, cytokines; SN, supernatants.

Immune outcomes were assessed in systemic and intestinal compartments of the dams at L14. The phagocytic activity and efficiency of blood monocytes and neutrophils was not modified after the supplementation with *Lactobacillus fermentum* CECT5716 (Figure 1A,B), nor were the proliferation of MLN lymphocytes and the subsequent production of intestinal cytokines (Figure 1C,D).

Despite that, we observed a different pattern of MLN immune populations between the LF and REF groups (Table 3). The proportion of cytotoxic T cells was reduced by ~3% in the dams receiving the probiotic compared to the control (*p* < 0.05). Such a decrease corresponded with a decline in the TCRαβ+ population (*p* < 0.05), whereas the TCRγδ+ displayed a similar proportion. Moreover, the expression of CD25, a marker of immune cell activation, was increased ~1% in both cytotoxic and helper T cells.

**Figure 2:**
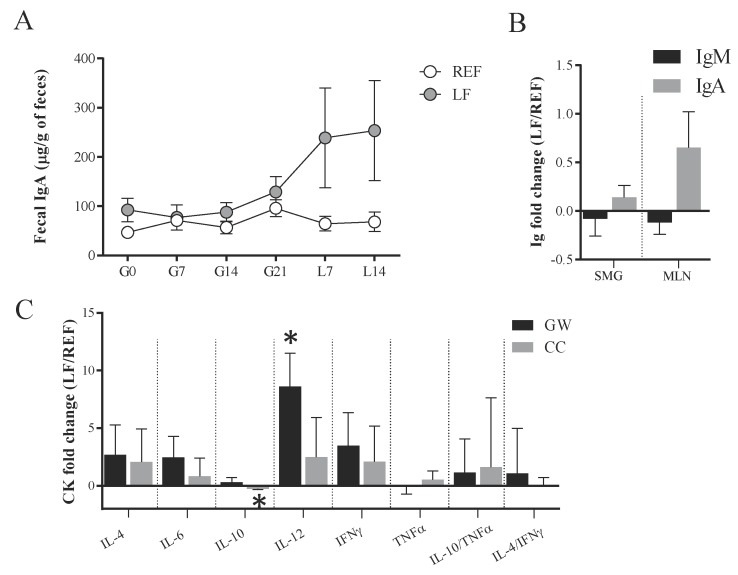
Mucosal immunoglobulins and cytokines of dams at L14. On the one hand, the evolution of (**A**) fecal IgA concentration was assessed throughout the study by ELISA. On the other hand, (**B**) the IgM and IgA were determined in submaxillary gland and mesenteric lymph nodes by ELISA at L14. Finally, (**C**) the concentration of cytokines in the gut wash and cecal content were analyzed by Luminex Technique at L14. Results are expressed as mean ± S.E.M. (*n* = 6/group). The fold change was calculated with respect to REF group. REF, reference group; LF, *Lactobacillus fermentum* CECT5716 group; Ig, immunoglobulins; SMG, submaxillary glands; MLN, mesenteric lymph nodes; CK, cytokines; GW, gut wash; CC, cecal content. * *p* < 0.05 compared to REF.

The fecal IgA concentration was analyzed throughout the study (Figure 2A). The amount of IgA was approximately 50–100 µg/g of feces during the gestation period in both groups (G0–G21), without showing differences between groups. However, after delivery (L7–L14), the levels of IgA were boosted in some of the LF group animals (>200 µg/g), although no statistical differences were observed when analyzing the whole group. In addition, tendencies were observed when calculating the area under the curve (*p* = 0.081) or calculating the increase of IgA from days G21 to L14 (*p* = 0.066).

IgA and IgM were also quantified in other mucosal structures, such as the submaxillary glands and MLNs, without showing differences in the fold change of either group (Figure 2B).

The quantification of intestinal cytokines was performed in the GW and CC (Figure 2C). The animals receiving the probiotic displayed a higher amount of IL-12 in the GW and a lower amount of IL-10 in the CC (*p* < 0.05).

The supplementation with the probiotic did not produce any changes in the fecal pH, fecal humidity or fecal weight throughout the study (Table 4).

### 3.4. Impact on the Ig and Cytokines of Dams and Pups

**Figure 3:**
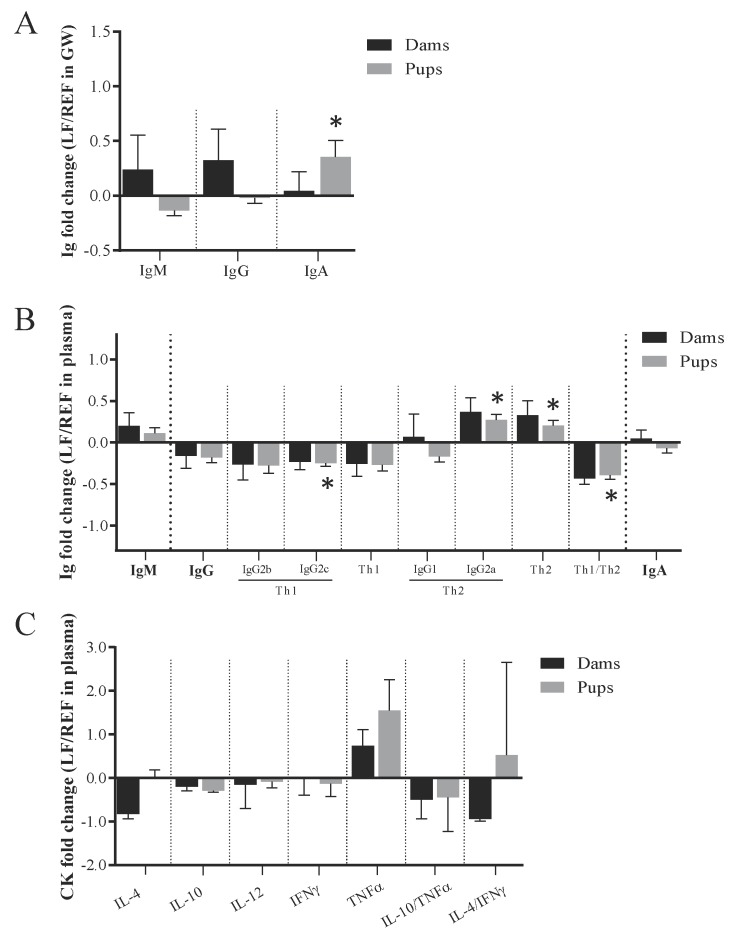
Systemic and intestinal immunoglobulins and cytokines of dams and pups at L14. (**A**) IgM, IgG and IgA were quantified in the gut wash by ELISA. (**B**) IgM, IgG and its subtypes, IgA and (**C**) cytokines were quantified in serum by Luminex Technique. Results are expressed as mean ± S.E.M. (*n* = 6 dams/group and *n* = 16 pups/group). The fold change was calculated with respect to REF group. REF, reference group; LF, *Lactobacillus fermentum* CECT5716 group; Ig, immunoglobulins; GW, gut wash; CK cytokines. * *p* < 0.05 compared to REF. # *p* = 0.05–0.1 compared to REF.

The intestinal Ig were quantified in GW at L14 (Figure 3A). The probiotic supplementation did not influence the amount of Ig in the dams at L14. Conversely, the pups showed a significant increase in the amount of IgA due to the probiotic administration (*p* < 0.05). Although this was not seen directly in the dams, the amount of IgA in the GW or in the feces of dams correlated positively with those levels found in the GW of the pups (*r* = 0.331 and 0.623, respectively, *p* < 0.05).

The systemic Ig were quantified in plasma at L14 (Figure 3B). The fold changes calculated between the LF and REF groups were similar both for dams and pups. On the one hand, the supplementation with *Lactobacillus fermentum* CECT5716 did not change the Ig profile of the dam, although a tendency to increase IgG2a was seen. On the other, the pups displayed significantly higher IgG2a (*p* < 0.05) and lower IgG2c concentrations. Overall, pups showed higher Th2-type Ig and consequently a reduction in the Th1/Th2 ratio (*p* < 0.05). Finally, the concentration of cytokines in serum was similar in both groups (Figure 3C).

### 3.5. Impact on the Plasma FA Profile of Dams and Pups

**Figure 4:**
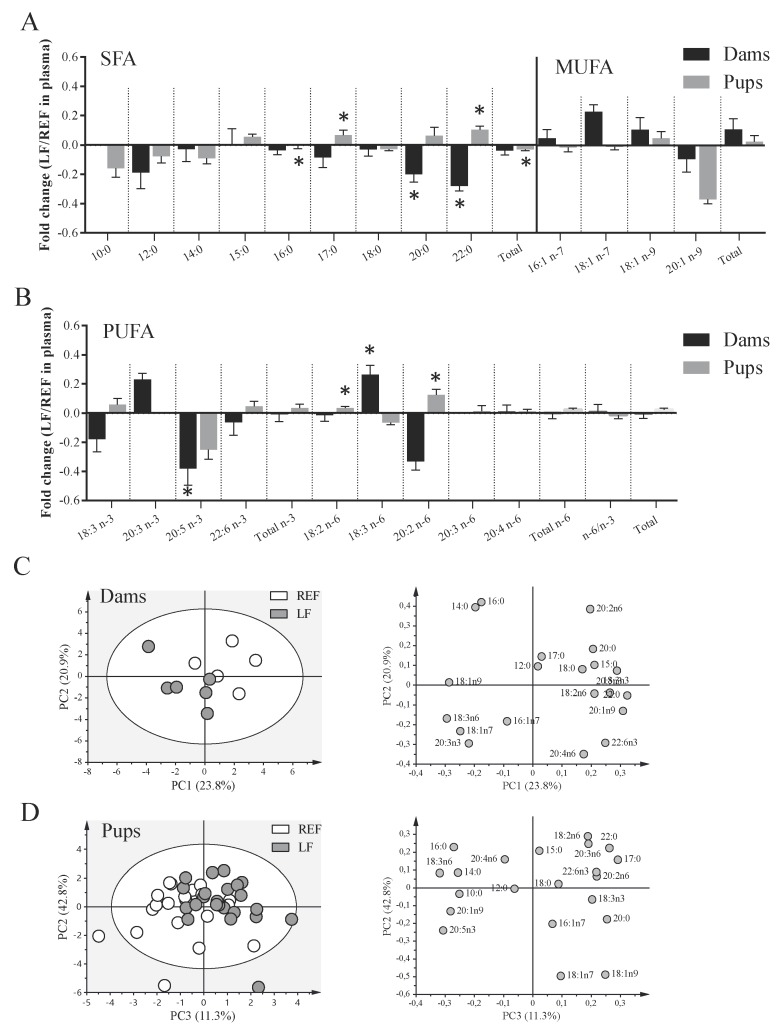
Fatty acid profile in serum of dams and pups at L14. (**A**) Saturated (SFA) and monounsaturated (MUFA) fatty acids, (**B**) polyunsaturated (PUFA) fatty acids were quantified by GC-FID. The analysis of principal components was performed for both (**C**) dams’ and (**D**) pups’ samples and represented in score and loading plots. Fatty acid data was mean centered and scaled to unit variance. The % of explained variance is presented in parentheses. Results are expressed as mean ± S.E.M. (*n* = 6 dams/group and *n* = 24 pups/group). The fold change was calculated in respect to REF group. REF, reference group; LF, *Lactobacillus fermentum* CECT5716 group. * *p* < 0.05 compared to REF.

The effects of *Lactobacillus fermentum* CECT5716 supplementation on the FA profile in plasma were assessed (Figure 4). The supplementation induced the reduction in the proportion of several FAs in the dams because negative fold changes in saturated FAs of 20 and 22 carbons and eicosapentaenoic acid (EPA, 20:5 *n-3*) could be observed (*p* < 0.05, Figure 4A,B). Conversely, an increase in γ-linolenic acid (GLA, 18:3 *n*-6) was detected (*p* < 0.05, Figure 4B). In addition, the probiotic supplementation in dams induced different effects on the plasma of pups. Their plasma was poorer in palmitic acid (PA, 16:0) and total saturated fatty acids (SFAs) (*p* < 0.05), but some minor FAs, such as those of 17 and 22 carbons, slightly increased (*p* < 0.05, Figure 4A). Moreover, the pups from supplemented dams displayed a higher proportion of linoleic acid (LA, 18:2 *n*-6) and eicosadienoic acid (EDA 20:2 *n*-6) (*p* < 0.05).

The analysis of principal components of the FAs revealed that the natural clustering of samples both in dams and pups was dependent on the supplementation, although the separation of the two experimental groups could be better observed in the dams (Figure 4C,D). The first three principal components explained 59.8% and 56.8% of the variance in the case of plasma FAs of dams and pups, respectively. According to the loading plots, it was observed that dam LF plasmas were positively correlated with 18:3 *n*-6, 18:1 *n*-9 and 18:1 *n*-7 contents while REF plasmas were positively correlated with 22:0, 18:3 *n*-3 or 20:5 *n*-3, among others. For pups, the separation of the LF group agreed with positive correlations with 17:0, 18:2 *n*-6 and 18:1 *n*-9, among others, while that of the REF group agreed with 16:0, 18:3 *n*-6 and 20:5 *n*-3, among others.

### 3.6. Impact on the Cecal Microbiota Composition of Dams and Pups

**Figure 5:**
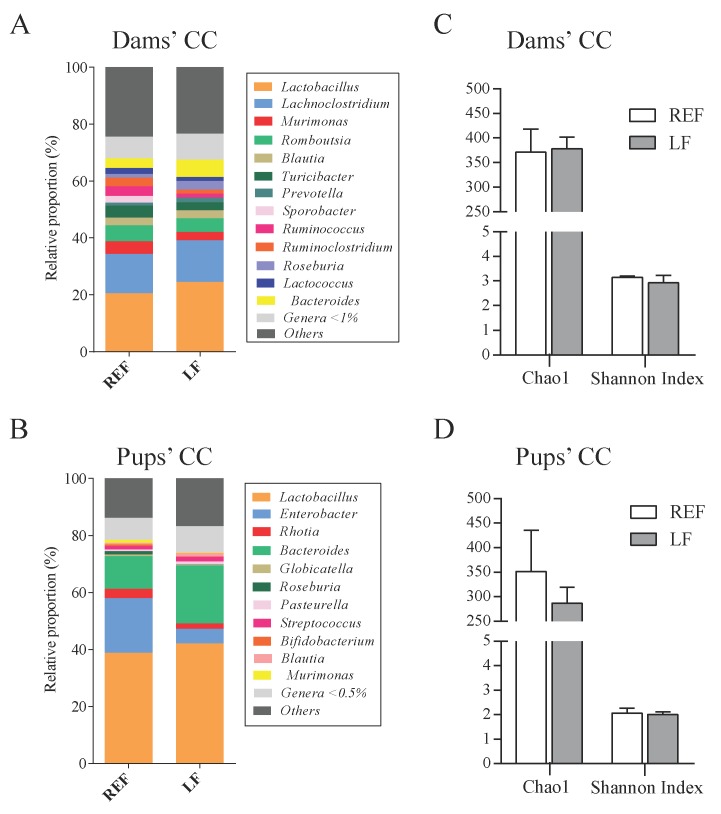
Cecal microbiota composition of dams’ and pups’ cecal content at L14. The main genera present in the cecal content are represented in stacked bars for (**A**) dams and (**B**) pups. Moreover, the richness of species and diversity of genera was analyzed by the calculation of Chao1 and Shannon Index, respectively, both for (**C**) dams and (**D**) pups. Results are expressed as mean ± S.E.M. (*n* = 3 animals/group). REF, reference group; LF, *Lactobacillus fermentum* CECT5716 group; CC, cecal content.

**Figure 6:**
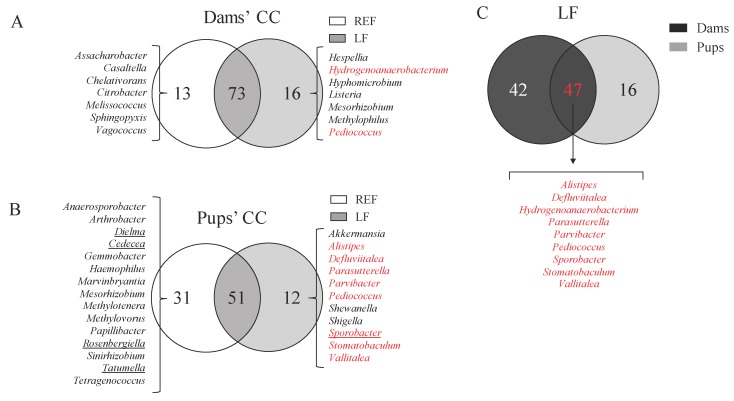
Venn diagrams of the genera present in the cecal content of dams and pups at L14. A bacterial group was considered as present by establishing a cutoff of two animals (out of the three samples analyzed) displaying proportions higher than 0.001%. Venn diagrams are represented for (**A**) dams’ CC, (**B**) pups’ CC and (**C**) the comparison of dams’ and pups’ CC for the LF group. Moreover, the genera highlighted in (**A**,**B**) correspond to those genera present in two or three animals in each group, and none of the animals in the other group. The animals underlined correspond to those which are present in 3/3 animals. The animals highlighted in red correspond to those which appear due to the supplementation with LF. Results are expressed as mean ± S.E.M. (*n* = 3 animals/group). REF, reference; LF, *Lactobacillus fermentum* CECT5716; CC, cecal content.

The intestinal microbiota composition was assessed by sequencing the CC bacterial DNA of dams and pups at L14 (Figure 5 and Figure 6). The group receiving the probiotic did not show any relevant differences in the proportion of phyla, families (data not shown) and genera (Figure 5A,B) or in the indicators of richness and diversity (Figure 5C,D). However, we could observe several qualitative differences through the analysis of Venn diagrams (Figure 6). In the dams’ CC, 73 out of 102 genera were present in both the LF and REF groups (~72%), whereas in the pups’ CC 59 out of 94 genera were present in both groups (~63%). The supplementation influenced the colonization of 16 genera (e.g., *Pediococcus* and *Listeria*) in dams and 12 in pups (e.g., *Sporobacter* and *Parvibacter*). In contrast, 13 and 31 genera found in the dams and pups of the REF group, respectively, were not present in the LF group (Figure 6A,B). Interestingly, 9 of the genera that were only present in the LF group were found both in pups and dams, such as *Pediococcus* and *Parvibacter* (Figure 6C).

## 4. Discussion

Pregnancy and breastfeeding periods are characterized by changes in the physiology of the mother that can influence the correct development and growth of the newborn. These changes may affect, for example, metabolic processes, the immune system and the microbiota composition. Therefore, pregnancy and breastfeeding can be considered as window periods in which to influence the health status of the mother and eventually to exert a beneficial impact on the offspring. In the present study we studied the effects of the supplementation with *Lactobacillus fermentum* CECT5716, a strain isolated from breast milk [40], in dams and pups.

At the end of the study *Lactobacillus fermentum* CECT5716 was detected in all CC of the dams and in the CC of some pups, which corresponded to pup siblings of three out of the six dams included in the study. These results suggest that the direct transmission of the probiotic from the mother to the pup can exist. Strains belonging to the species *B. breve*, *B. longum*, *E. faecalis*, *L. fermentum*, *L. gasseri*, *L. salivarius,* and *S. epidermidis* have evidenced transmission through breast milk to the infant gut [40,61,62,63]. In this context, previous studies in our laboratory found that oral administration of *Lactobacillus fermentum* CECT5716 during pregnancy and gestation led to its presence in rat breast milk [50]. In addition, Arroyo et al. were able to isolate *Lactobacillus fermentum* CECT5716 in breast milk of women suffering from mastitis who received the probiotic [45]. Thus, altogether the results herein are compatible with the entero-mammary route, where physiological bacterial translocation from the digestive tract has been proposed as a source of bacteria for the mammary gland and breast milk, making possible their subsequent transmission to the offspring [15,16,64]. The transfer of this strain and other bacteria through coprophagy from dam to pup can be discarded because the rats do not start ingesting maternal feces until at least L16 [52].

The administration of the probiotic strain did not modify the weight of the dams or pups throughout the study nor the weight of the organs analyzed. In addition, no adverse effects were visually detected due to the supplementation. These results suggest that the administration of *Lactobacillus fermentum* CECT5716 in pregnant and lactating rats is safe and well tolerated and does not impact negatively on their offspring. Several studies found no differences in the weight, head circumference and growth rate of infants receiving a formula supplemented with this probiotic [43,44]. Similarly, a study by Lara-Villoslada et al. reported no effects of this strain on the weight gain of mice or the weight of their liver, spleen, thymus, heart, and kidney [65]. In contrast, in the context of stress-induced barrier dysfunction, rat pups supplemented with this probiotic displayed significantly increased body length and weight and greater small and large intestine lengths compared to control pups [66]. 

We aimed at assessing the impact of the probiotic in different systemic and intestinal immune variables of the mother that might have a key role in early life. While cellular acquired immunity is depressed during pregnancy, innate immunity, such as phagocytic activity has been reported to be increased [67]. In addition, breast milk is rich in phagocytes with microbiocidal activity [68]. Díaz-Ropero et al. reported increased phagocytic activity of circulating blood leucocytes in 6-week-old mice receiving this probiotic strain [48]. Nevertheless, herein, dams supplemented with *Lactobacillus fermentum* CECT5716 did not display higher circulant monocytes and neutrophils with phagocytic activity compared to the control group. Neither did they show increased phagocytic efficiency, although the mean value was visually higher in the LF group. This may be explained by differences in the probiotic dose, the animal model or the physiological condition.

Hypothetical translocation of bacteria to the mammary gland has been suggested to be orchestrated by phagocytes that carry the bacteria through the MLNs; therefore, we presumed that MLNs would be a suitable compartment to observe probiotic-derived effects. With this in mind, we evaluated the functionality of MLN lymphocytes but failed to detect changes in their proliferation or production of cytokines. In contrast, we observed changes in the proportion of the immune cells in this compartment. The dams supplemented with *Lactobacillus fermentum* CECT5716 displayed a lower proportion of TCRαβ+ cytotoxic T cells. Although more experiments should be performed to decipher how this is produced, such a decrease might be attributed to greater migration of cytotoxic T cells to the mucosa. Indeed, in the present study the increase in T cell activation, assessed by the expression of CD25+, is in accordance with this hypothesis. In this context, an in vitro study by Pérez-Cano et al. evidenced that the co-culture of human peripheric blood mononuclear cells with *Lactobacillus fermentum* CECT5716 induced a higher activation status of T cells, assessed by both CD69 and CD25 markers [49]. Alternatively, an apparent increase in the proportion of B cells might also explain a lower relative proportion of T cell subsets. The administration of probiotics has been proposed to increase intestinal IgA production in the lamina propria by the induction of B cells in the Peyer’s patches and MLNs [69,70]. Although no significant differences in the concentration of IgA were observed in the present study, we did observe a tendency to increased fecal and MLN IgA of the mother in the LF group, suggesting a possible mucosal humoral immune enhancement. 

In addition, we found a higher concentration of gut IL-12 after supplementing with the probiotic. In this regard, the capture of probiotics by macrophages and dendritic cells has been shown to induce the production of cytokines, such as TNFα and IFNγ, in order to enhance epithelial cell stimulation and trigger the crosstalk in the gut immune system [69]. Accordingly, *Lactobacillus fermentum* CECT5716 administration increased IL-12 production in spleen-derived lymphocytes of healthy mice pups [48] or rat pups suffering stress-induced intestinal barrier dysfunction [66]. 

We also aimed to assess immune variables measurable in both the dam and the pup in order to investigate whether the effects are transmittable or have an impact on the offspring. Interestingly, we detected increased concentration of intestinal IgA in those pups whose mothers were supplemented with the probiotic during gestation and lactation (14 days), but not in the mothers themselves. Because the immune system is still immature, most IgA found in the intestine of the pup comes from the breast milk of the mother. In this context, previous studies in our laboratory evidenced that supplementation of dams with *Lactobacillus fermentum* CECT5716 was able to increase the amount of IgA in breast milk [50], which certainly correlates with the increase we find here in the pups. Furthermore, administration of *Lactobacillus fermentum* CECT5716 directly to infants did not show increased gut IgA levels [42], suggesting that the administration of the probiotic to the mother, who has a mature immune system, would be better in terms of providing enhanced protection through IgA to the newborn. In contrast, Díaz-Ropero et al. found increased IgA concentration in feces of mice consuming the same probiotic, although these were 6 weeks old and therefore they probably displayed a more mature immune system [48].

The profile of plasmatic IgG was modified due to the probiotic supplementation. In particular, the fold changes with respect to the control group displayed a similar mean value for all IgG when comparing the dams and pups, indicating that the same effects elicited on the systemic Ig composition of the mother were reproduced in the pups. Overall, the type of IgGs were biased towards less Th1-type (IgG2c) and more Th2-type (IgG1 and IgG2a). Previous studies in our laboratory also evidenced that supplementation with *Lactobacillus fermentum* CECT5716 induced a trend towards a lower Th1/Th2 Ig pattern ratio in breast milk [50]. In fact, it is feasible to think that the systemic IgG composition in the pup reproduces the same pattern in breast milk, because they are absorbed in the rodent’s gut [71,72]. However, the administration of *Lactobacillus fermentum* CECT5716 directly to infants did not influence their composition of plasma IgG [48], once again supporting the idea that better immune outcomes might be achieved when administering the strain to the mother. Other researchers also detected modulation of systemic IgG after the oral administration of other lactobacilli strains in mice [73,74,75].

Recent studies suggest that probiotic administration may impact the lipid profile by affecting their absorption or metabolism [76,77,78]. With this in mind, we also quantified the plasmatic FA profile of the dams and pups. We observed different effects due to the probiotic supplementation, because while dams showed lower amounts of EPA and higher GLA levels, pups displayed less PA and total SFA and higher LA. On the one hand, the impact on the mother may be through direct intestinal or systemic effects exerted by the probiotic, while on the other hand the impact on the pups is probably coming from the lipid composition of breast milk. In fact, these results in pup plasma agree with previous studies in our laboratory that evidenced that supplementation with *Lactobacillus fermentum* CECT5716 in pregnant and lactating mothers led to a lower proportion of PA and increased LA and α-linolenic acid (ALA, 18:3 *n-3*) in milk [50]. Therefore, the increase in LA of pups, an essential FA, could be derived from its increased intake from breast milk. 

Finally, the cecal microbiota abundance of the dams and pups at the end of the study did not experience significant changes due to the probiotic supplementation. In addition, previous studies in our laboratory also reported no effects in the microbiota of breast milk [50]. Accordingly, an infant formula supplemented with *Lactobacillus fermentum* CECT5716 did not show differences in the pattern of fecal microbiota when administered directly to infants [42,43]. As one of the limitations in the analysis of the microbiota in this study is the sample size, the possibility that changes due to supplementation can occur in the pregnancy or early lactation periods cannot be ruled out. However, several qualitative differences were found between the LF and REF group because some genera were exclusively found in each group and, interestingly, some of these are shared by mother-pup pairs. Future studies should be conducted in order to establish the biological relevance of the dampened colonization of *Cedecea*, *Dielma*, *Rosenbergiella,* and *Tatumella* and the colonization of *Sporobacter* in the pups of the LF group. This might suggest that probiotic supplementation may be influencing the colonization of the infant with bacteria from the mother, independently if they are new colonizers due to the administration of LF or not.

## 5. Conclusions

Overall, the administration of *Lactobacillus fermentum* CECT5716 in rats during pregnancy and lactation periods was safe and well tolerated. In addition, the probiotic was detected in the CC of all dams and in the CC of several pups, indicating the possibility of an entero-mammary route. However, the observed effects did not seem to be linked directly to probiotic arrival to the pup. Immunomodulation in the dam was observed at the end of the study, by changing the proportions of MLN lymphocyte T cell subsets and their activation. Moreover, changes in systemic IgG and lipid profile were seen both in dams and pups. The effects on the pups, and especially the increase in intestinal IgA that was found, seem to be a reflection of the breast milk composition. The administration of the probiotic to the mothers, with a mature immune system, rather than to the pups, with an immature immune system, seems to be more effective in terms of achieving increased immune defenses in the newborn. Therefore, the administration of *Lactobacillus fermentum* CECT5716 in early life has a beneficial impact on the lipid profile and immune system of the mother and her offspring.

## Figures and Tables

**Table 1:** Detection of *Lactobacillus fermentum* CECT5716 in the cecal content (CC) of dams and pups.

	REF						LF					
	1	2	3	4	5	6	7	8	9	10	11	12
**Dams’ CC**												
**Pups’ CC**							50%	25%	25%			

Results are expressed as mean ± S.E.M. (*n* = 4–6/group). Grey squares represent samples with positive detection (including the percentage of detection) and white squares samples with no detection. CC, cecal content; REF, reference group; LF, *Lactobacillus fermentum* CECT5716 group.

**Table 2:** Growth and relative organ size of dams and pups.

		Dams		Pups	
		REF	LF	REF	LF
* Body **Weight**	**G21/L4**	406.24 ± 18.26	395.47 ± 12.12	9.91 ± 0.20	9.90 ± 0.16
	**L14**	345.57 ± 7.96	325.38 ± 11.80	28.94 ± 0.40	28.85 ± 0.59
**Size**	**BMI (g/cm^2^)**	0.71 ± 0.04	0.68 ± 0.03	0.34 ± 0.01	0.34 ± 0.01
	**Lee Index (g0.33/cm)**	318.04 ± 8.19	315.19 ± 5.69	331.83 ± 1.30	332.90 ± 1.42
* Organ Weights	**Stomach**	0.94 ± 0.25	0.67 ± 0.02	0.65 ± 0.01	0.69 ± 0.05
	**Small intestine**	3.93 ± 0.10	3.85 ± 0.06	3.06 ± 0.03	3.16 ± 0.03
	**Cecum**	0.45 ± 0.02	0.48 ± 0.02	0.15 ± 0.01	0.14 ± 0.01
	**Large intestine**	0.63 ± 0.05	0.57± 0.03	0.33 ± 0.05	0.28 ± 0.01
	**Spleen**	0.21 ± 0.01	0.24 ± 0.01	0.49 ± 0.01	0.52 ± 0.01
	**Thymus**	0.13 ± 0.01	0.13 ± 0.01	0.46 ± 0.01	0.57 ± 0.09
	**Liver**	4.02 ± 0.15	4.16 ± 0.10	3.19 ± 0.04	3.02 ± 0.07
	**Kidneys**	0.57 ± 0.01	0.60 ± 0.02	1.15 ± 0.01	1.16 ± 0.01
	**Brain**	0.42 ± 0.06	0.41 ± 0.07	3.55 ± 0.06	3.72 ± 0.07
	**Uterus**	0.10 ± 0.01	0.10 ± 0.01	-	-
	**Ovaries**	0.05 ± 0.01	0.05 ± 0.01	-	-
	**Heart**	0.34 ± 0.01	0.36 ± 0.01	-	-
**^#^ Organ Length**	**Small intestine**	25.32 ± 2.43	23.25 ± 3.24	5.55 ± 0.18	135.88 ± 2.36
	**Cecum**	1.79 ± 0.06	1.92 ± 0.13	-	-
	**Large intestine**	5.63 ± 0.45	5.55 ± 0.18	-	-

Results are expressed as mean ± S.E.M. (*n* = 6 dams/group and 48 pups/group). * Body weight expressed in g and organ weights expressed in 100 g of tissue/g of body, ^#^ Organ length expressed in cm of tissue/g of body. BMI, body mass index; REF, reference group; LF, *Lactobacillus fermentum* CECT5716 group, G21 (dams, before birth); L4 (pups, day 4 of life).

**Table 3:** Characterization of mesenteric lymph node cells phenotype of dams.

	REF	LF
**%B cells (CD45RA+)**	26.93 ± 3.31	32.75 ± 5.02
CD62L+ (%)	78.62 ± 4.35	78.82 ± 3.87
CD25+ (%)	0.70 ± 0.12	0.86 ± 0.31
**%Th cells (CD4+ TCRαβ+)**	43.37 ± 1.36	40.23 ± 1.87
CD62L+ (%)	70.91 ± 1.53	70.98 ± 1.89
CD25+ (%)	6.81 ± 0.34	8.00 ± 0.43*
αE integrin (%)	4.06 ± 0.76	5.70 ± 1.63
**%Tc cells (CD8+ TCRαβ+ and TCRγδ+)**	20.54 ± 0.51	17.15 ± 1.14*
CD62L+ (%)	85.28 ± 1.70	86.81 ± 0.89
CD25+ (%)	2.58 ± 0.18	3.38 ± 0.68*
αE integrin (%)	5.13 ± 1.26	7.07 ± 2.60
**TCRαβ+ (CD8+ TCRαβ+ NK-) (%)**	19.09 ± 0.49	15.57 ± 1.12*
**TCRγδ+ (%)**	1.69 ± 0.14	1.89 ± 0.15
CD8- TCRγδ+ (%)	0.25 ± 0.02	0.31 ± 0.03
CD8+ TCRγδ+ (%)	1.45 ± 0.13	1.58 ± 0.13
CD8αα+ TCRγδ+ (%)	0.14 ± 0.02	0.16 ± 0.02
CD8αβ+ TCRγδ+ (%)	1.31 ± 0.11	1.42 ± 0.12
**%NKT cells (TCRαβ+ NK+)**	2.49 ± 0.16	2.45 ± 0.26
**%NK cells (TCRαβ- NK+)**	2.11 ± 0.11	2.42 ± 0.19

Results are expressed as mean ± S.E.M. (*n* = 6/group). * *p* < 0.05 compared to REF. Th, T helper; Tc, T cytotoxic; REF, reference group; LF, *Lactobacillus fermentum* CECT5716 group.

**Table 4:** Evolution of pH, humidity and weight of the dams’ feces throughout the study.

		G0	G7	G14	G21	L7	L14
**Fecal pH**	**REF**	5.88 ± 0.05	6.20 ± 0.10	6.09 ± 0.12	6.08 ± 0.06	6.48 ± 0.08	6.47 ± 0.05
	**LF**	6.07 ± 0.14	6.48 ± 0.19	6.40 ± 0.26	6.43 ± 0.14	6.50 ± 0.16	6.11 ± 0.12
**Fecal humidity (%)**	**REF**	-	50.59 ± 3.33	55.91 ± 1.24	57.87 ± 2.10	62.97 ± 1.40	65.41 ± 0.78
	**LF**	-	53.44 ± 5.02	58.68 ± 2.81	58.53 ± 1.07	65.19 ± 2.39	62.07 ± 4.36
**Fecal weight (mg)**	**REF**	-	259.4 ± 39.6	144.0 ± 19.2	249.3 ± 40.1	421.8 ± 53.9	452.6 ± 49.0
	**LF**	-	306.83 ± 50.2	278.0 ± 59.6	185.5 ± 16.5	369.3 ± 16.6	329.8 ± 32.6

Results are expressed as mean ± S.E.M. (*n* = 6/group). G, gestation; L, lactation; REF, reference group; LF, *Lactobacillus fermentum* CECT5716 group.

## Supplementary Materials

The following are available online at https://www.mdpi.com/2073-4409/9/3/575/s1. Table S1: Assessment of the matrix effect on the quantification of immunoglobulins by ELISA.
