# Supplementary material for: Lactobacillus fermentum CECT5716 Supplementation in Rats during Pregnancy and Lactation Impacts Maternal and Offspring Lipid Profile, Immune System and Microbiota
Source: Cells. 2020 Feb 28;9(3):575. doi: 10.3390/cells9030575 (PMC7140451; doi:10.3390/cells9030575)
Supplement: Supplementary file 1 [file cells-09-00575-s001.pdf]

cells

Article: Supplementary Data

Supplementary Table S1. Assessment of the matrix effect on the quantification of immunoglobulins by ELISA.

	IgM		IgG		IgA	
	dilution	% recovery	dilution	% recovery	dilution	% recovery
**Fecal homogenate**	-	-	-	-	1/10	82.1 ± 0.7
**GW (dams)**	1/10	99.1 ± 1.1	1/500	89.8 ± 2.6	1/100	93.8 ± 13.0
**GW (pups)**	1/30	98.7 ± 5.6	1/2000	93.3 ± 2.7	1/200	105.3 ± 0.8
**MLN homogenate**	1/20	98.4 ± 4.9	-	-	1/80	90.0 ± 2.0
**SMG homogenate**	1/10	88.3 ± 11.3	-	-	1/40	102.4 ± 5.8

A spike recovery test was performed to investigate if the concentration-response relationship was similar in the calibration curve using the kit diluent and using each of the sample diluent. Samples were spiked with three different concentrations of the standard within the linear part of the calibration curve. The percentage of recovery was calculated as: (Measured concentration of the spiked sample – Measured concentration of the neat sample)/(Theoretical concentration of the spike) x 100. Results are expressed as mean ± S.E.M. Gut wash (GW), immunoglobulin (Ig), mesenteric lymph nodes (MLNs) and submaxillary gland (SMG).
